# *In situ* 3D-patterning of electrospun fibers using two-layer composite materials

**DOI:** 10.1038/s41598-020-64846-z

**Published:** 2020-05-14

**Authors:** R. L. Creighton, J. Phan, K. A. Woodrow

**Affiliations:** Department of Bioengineering, University of Washington, Seattle, WA 98195 USA

**Keywords:** Biomaterials, Biomedical engineering, Materials for devices

## Abstract

Polymeric electrospun nanofibers have extensive applications in filtration, sensing, drug delivery, and tissue engineering that often require the fibers to be patterned or integrated with a larger device. Here, we describe a highly versatile *in situ* strategy for three-dimensional electrospun fiber patterning using collectors with an insulative surface layer and conductive recessed patterns. We show that two-layer collectors with pattern dimensions down to 100-micrometers are easily fabricated using available laboratory equipment. We use finite element method simulation and experimental validation to demonstrate that the fiber patterning strategy is effective for a variety of pattern dimensions and fiber materials. Finally, the potential for this strategy to enable new applications of electrospun fibers is demonstrated by incorporating electrospun fibers into dissolving microneedles for the first time. These studies provide a framework for the adaptation of this fiber patterning strategy to many different applications of electrospun fibers.

## Introduction

Electrospun fibers are a unique material with broad capabilities in filtration, sensing, drug delivery, and tissue engineering due to the versatility of materials that can be processed. Because of their interconnected pores, which create a tortuous path for particles, electrospun fibers have been developed for commercial use in air and liquid filtration^1–4^. This filtration function combined with mechanical strength and breathability has also made electrospun fibers ideally suited to act as protective textiles for chemical or biological toxins^5^. Electrospun polymers with fluorescent or colorimetric activity have also been used as sensors for various environmental hazards like mercury ions and health hazards like organic solvents^6–8^. Electrospun fibers are useful in many different biological applications including tissue engineering and drug delivery because of the wide range of biocompatible materials that can be electrospun and the variety of strategies for incorporating physicochemically diverse agents^9–12^. Additionally, the mechanical and chemical properties of electrospun polymeric fibers can be simultaneously engineered through polymer selection, fiber alignment, and biologic incorporation^13^. These engineered electrospun fibers have been a useful tool for supporting cell viability in engineered tissues and for studying the effect of mechanical and chemical cues on cell phenotype^14–16^.

For these applications, electrospun fibers need to be patterned on various length scales for optimal function or for device integration. This is often achieved by electrospinning fibers in large sheets that are then mechanically cut into pieces with dimensions down to 5 mm and positioned within devices^17,18^. Bulk mechanical patterning has been used to layer electrospun fibers between microfluidic channels for portable and lightweight dialysis systems^17^. Fibers have also been cut and layered within the core of stimuli-responsive hydrogels for controlled drug delivery, but in this application the mechanical fiber patterning strategy resulted in uncontrolled diffusion at the device edges^18^. Mechanical fiber patterning is not generalizable because it can lead to poor control over device dimensions at small length scales^18^, and it is not feasible for complex device designs such as micro-scale grids or microfluidic channels containing discreet micro-scale fiber regions^8,19,20^. Furthermore, mechanical patterning and integration strategies can lead to fiber deformation and alteration of the fiber mat porosity, which is critical to its function for filtration, drug delivery, and tissue engineering^1^. Therefore, there is a need for alternative fiber patterning strategies that preserve fiber structure and function, improve fiber function in existing applications, and enable new applications.

Several alternative fiber patterning strategies currently exist. Processes like photolithography and ultraviolet and femtosecond laser ablation have enabled precise fiber integration within microfluidic channels^19,21^, and have been used to increase cell infiltration and isolate cells within fiber mats^22–24^. However, many of these patterning processes are incompatible with fibers intended for biological applications, and these processes can alter polymer fiber molecular weight, reduce tensile strength, and introduce debris into the final fiber structure^22,24^. Because of these limitations, *in situ* fiber patterning strategies like near field electrospinning and electrostatic manipulation are better suited for applications requiring preservation of fiber morphology and biological reagents^25–28^. Near field electrospinning enables precise control over the deposition of single fibers to generate pattern dimensions between 25 μm and 1 mm, which is useful for characterization of fiber properties such as piezoelectricity^29^, but the process is low throughput and not ideal for large scale devices^25^. Electrostatic based fiber patterning using electric field lenses or patterned collectors has high throughput, biological compatibility, and preserves fiber morphology. However, electric field lenses can only control fiber deposition on the centimeter scale^30^, and existing patterned collectors lack versatility because the fiber patterning effect requires a particular polymer or a specific pattern design and dimension^26–28^. Taken together, the current state of the field suggests a need for an *in situ* fiber patterning strategy with more versatility in terms of fiber material, pattern complexity, and pattern dimensions.

Here, we describe a highly versatile *in situ* strategy for three-dimensional electrospun fiber patterning based on two-layer collectors with an insulative surface and conductive recessed patterns. Using finite element method simulations, we evaluated the effect of various collector design factors on the predicted fiber patterning. Key findings from these simulations were then verified experimentally. This strategy is compatible with different fiber materials and pattern dimensions, which will enable use in a broad range of future applications. To demonstrate one potential application of this new fiber patterning strategy, electrospun fibers were integrated into dissolving microneedles for the first time.

## Results and Discussion

### Rapid fabrication of complex electrospun fiber patterns using two-layer collectors

Previous studies have demonstrated that electrospun fibers are extremely sensitive to minor changes in electric field and collector dielectric properties^30^. Fiber deposition has been focused into centimeter scale spot sizes through manipulation of the electric field^30^, and fiber collection density has been modified on the micrometer scale by micropatterned collectors^26,27^. However, no single *in situ* electrospun fiber patterning strategy has demonstrated generalizability from micrometer to centimeter length scales for a variety of relevant fiber materials. Additionally, existing *in situ* fiber patterning strategies are not capable of patterning in three dimensions on a range of length scales, which could prove valuable for integrating fibers within drug delivery systems, sensors, or microfluidic channels^17,18^.

**Figure 1:**
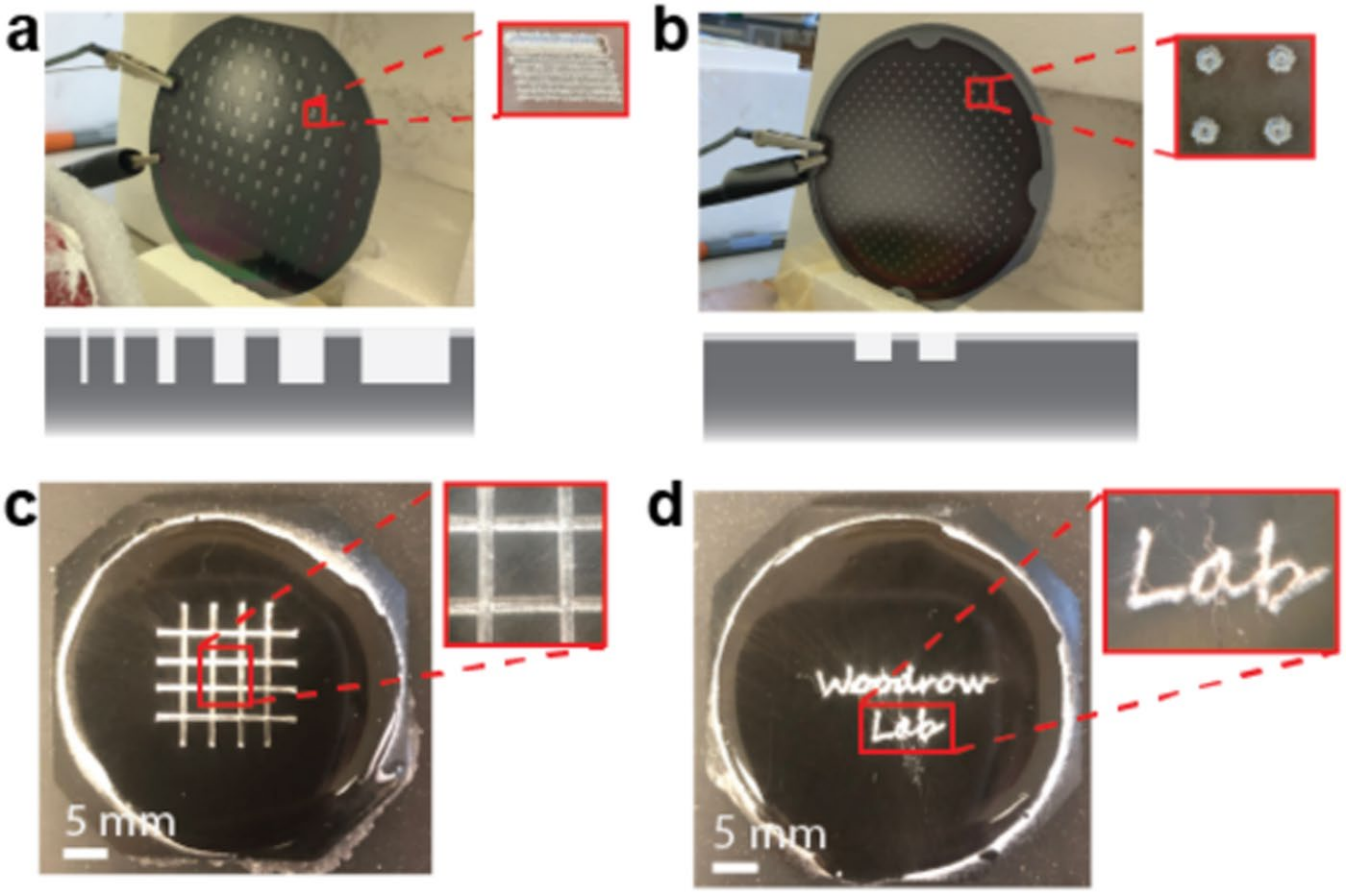
Rapid fabrication of complex electrospun fiber patterns using two-layer collectors. (**a**) Trench and (**b**) doughnut micropatterns were created in silicon using traditional microfabrication techniques. Schematic (not to scale) of the collector cross-sections depict the trench pattern depth (150 μm) and range of widths (500 to 5 μm) and the doughnut pattern depth (50 μm). The 2 μm thick photoresist layer was left on the silicon wafers to act as the insulative surface layer. (**c**,**d**) Patterns that were approximately 500 μm wide and 500 μm deep were created in a carbon black-PDMS composite material with an approximately 600 μm thick PDMS insulative surface layer using a CO_2_ laser cutter. On all of these collectors, polyvinyl alcohol (PVA) fibers deposited densely in the micropatterns compared to the collector surface.

We investigated the ability of collectors with an insulative surface layer and conductive recessed patterns to generate three-dimensional patterned fibers on a range of length scales. To evaluate this concept, we first created micropatterned collectors in silicon. Trench patterns with 150-μm depth and doughnut patterns with 50-μm depth were created in silicon wafers using standard photolithographic patterning and deep reactive ion etching. The approximately 2-μm thick polymeric photoresist was left on the wafer after etching, creating a two-layer patterned collector with an insulative surface layer (Fig. 1a,b). Poly(vinyl alcohol) (PVA) fibers were readily deposited over the majority of the collector, and fibers deposited more densely in the silicon patterns than on the photoresist-coated collector surface (Fig. 1a,b). We expected that the insulative photoresist layer caused an increase in electric potential at the collector surface, leading to fiber repulsion from the surface and deposition in the conductive recessed patterns.

To explore the constraints of this fiber patterning strategy on a larger scale, we created poly(dimethyl siloxane) (PDMS)-based collectors. PDMS is easy to manipulate on a range of length scales using either additive or subtractive methods^31–33^. Additionally, PDMS can be made conductive by incorporating high concentrations of conductive particulates such as carbon black^34^. We first created a conductive mixture of carbon black and PDMS (C-PDMS), then added an approximately 600-μm thick layer of insulative PDMS on the surface. Use of this material enabled rapid fabrication of complex patterns on the scale of hundreds of micrometers using a CO_2_ laser. Similar to the silicon collectors, PVA fibers deposited more densely within the patterns compared to the collector surface (Fig. 1c,d). Together, these preliminary experiments demonstrated that patterned conductive collectors with an insulative surface layer could be used to generate patterned electrospun fibers on a range of length scales.

### Finite element method simulations following statistically designed experiments for collector geometry designs

**Figure 2:**
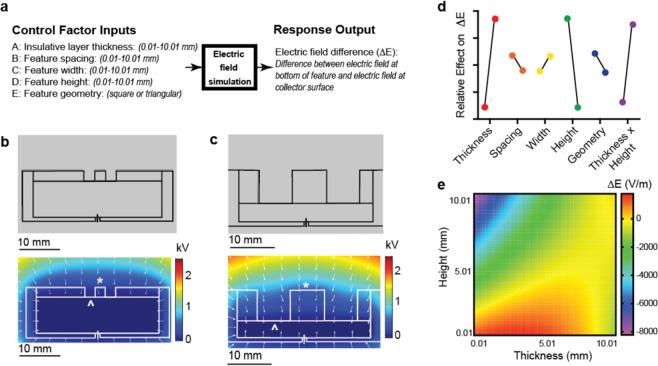
Finite element method simulations following statistically designed experiments for collector geometry designs. (**a**) P-diagram of the design of experiments, including the five different control factor inputs and the primary response output. Factors were varied from 2.51 mm to 7.51 mm in the initial full factorial design, then were varied from 0.01 mm to 10.01 mm for the central composite design. Representative images from two distinct simulations for square feature geometry with (**b**) 2.51 mm width, height, spacing, and thickness, and (**c**) 7.51 mm width, height, spacing, and thickness. These images show the starting geometry, the calculated electric potential (color gradient, scale in kV), and electric field (arrow vectors). Annotations indicate the location of the collector surface (*) and bottom of the pattern (^) used to calculate the ΔE response output. (**d**) Means plot showing the relative effect of each factor at each level. The largest effect was observed for the insulative layer thickness, feature height, and the interaction of these two factors. (**e**) The heat map of ΔE from the quadratic model indicates that ΔE is maximized by limiting feature height to 5 mm and increasing the insulative layer thickness proportionally with increasing feature height.

We sought to evaluate the design space of our fiber patterning strategy using a series of two-dimensional finite element method simulations. The first set of simulations followed a two-level, five factor, full factorial design to determine collector design factors that would have the most significant effect on electrospun fiber patterning. Factors of interest included insulative layer thickness and feature spacing, width, height and geometry (Fig. 2a). The primary response output was the difference between the electric field at the bottom of the pattern and the electric field at the collector surface (ΔE). Assuming the deposition of the electrospun fibers is primarily driven by the force of the electric field, a ΔE greater than 0 would predict more fiber deposition in the patterns compared to the collector surface.

Each simulation calculated the potential and electric field for geometries, materials, and electrostatic conditions matching actual experimental setups to be used in later studies (Fig. 2b,c). Two sample simulations for small scale (2.5 mm width, height, spacing, and thickness) and large scale (7.5 mm width, height, spacing, and thickness) square patterns computed a ΔE of -143 V/m for the small scale pattern and a higher ΔE of 105 V/m for the large scale pattern (Fig. 2b,c). To determine the factor or factors that were driving this result, the main effect size for each factor was calculated. The main effect for each factor is defined as the difference between the average ΔE at its high and low levels holding all other factors constant. For this set of experiments, the insulative layer thickness and feature height had the largest effect on ΔE (effect size = 1,615 and −1,632 V/m, respectively) (Fig. 2d). These results indicate that an increase in insulative layer thickness or a decrease in feature height would result in an increased ΔE, which predicts increased fiber deposition in the pattern. Calculation of two- and three-factor interactions showed that only the interaction of insulative layer thickness and feature height had an appreciable effect on ΔE (effect size = −1,424 V/m). Because these two factors interact, their effect should be considered by the interaction term rather than the individual main effects. Statistical analysis showed that all of the evaluated factors and the thickness-height interaction factor had a significant effect on ΔE (Table 1). However, it was also clear from this analysis that ΔE is primarily determined by the thickness-height interaction. These results suggest that appropriate selection of just feature height and insulative layer thickness could yield electrospun fiber patterns of a variety of widths, spacing, and shape with high selectivity.

**Table 1:** Statistical analysis of finite element method simulations.

Full Factorial Design					
	*Sum of Squares*	*df*	*Mean Square*	*F Value*	*p-value, Prob* > *F*
*Model*	6.05E7	6	1.00E7	228	1.37E-20
*A-PDMS thickness*	2.08E7	1	2.08E7	472	9.34E-18
*B-Spacing*	6.18E5	1	6.18E5	14.0	9.56E-04
*C-Width*	6.39E5	1	6.39E5	14.4	8.15E-04
*D-Height*	2.13E7	1	2.13E7	482	7.26E-18
*E-Shape*	8.59E5	1	8.59E5	19.4	1.70E-04
*AD*	1.62E7	1	1.62E7	367	1.81E-16
**Central Composite Design**					
*Model*	5.09E7	7	7.28E6	58.2	9.78E-13
*A-PDMS Thickness*	1.32E7	1	1.32E7	105	7.18E-10
*B-Spacing*	1.91E5	1	1.91E5	1.53	0.229
*C-Width*	1.52E5	1	1.52E5	1.21	0.281
*D-Height*	2.70E7	1	2.70E7	216	7.16E-13
*AD*	7.13E6	1	7.13E6	57.0	1.51E-07
*A*^2^	3.12E6	1	3.12E6	24.9	5.29E-05
*D*^2^	162	1	162	0.00129	0.972

To further explore factor interactions and to evaluate a larger range of pattern dimensions, we conducted another set of simulations following a central composite experimental design. Shape was omitted as a factor to simplify the design and because the full factorial design indicated that it was not one of the main factors affecting ΔE. The results were similar to the results from the full-factorial design, with the largest and most significant effects observed for insulative layer thickness, feature height, and the interaction of these two factors (Table 1). A quadratic model based on the results was then used to construct a heat map of the interaction between insulative layer thickness and feature height for intermediate values of spacing (5 mm) and width (5 mm). This heat map indicated that to maximize ΔE for the dimensions evaluated here, feature height should be limited to 5 mm and insulative layer thickness should be increased proportionally as feature height increases (Fig. 2e). Importantly, ΔE can be increased for feature heights greater than 5 mm by further increasing the insulative layer thickness beyond the range of these simulations. For example, ΔE of 2,581 V/m was calculated for a triangular feature with 5.01 mm width, 15 mm height, 5.01 mm spacing, and 20 mm insulative layer thickness. Applications that require larger feature sizes will require additional simulations centered around the dimensions of interest to ensure accurate prediction of ΔE. Together these simulations suggest that this fiber patterning strategy can be used over a wide range of length scales.

### Rapid and inexpensive collector prototyping by subtractive and additive processes using a PDMS-based composite material

**Figure 3:**
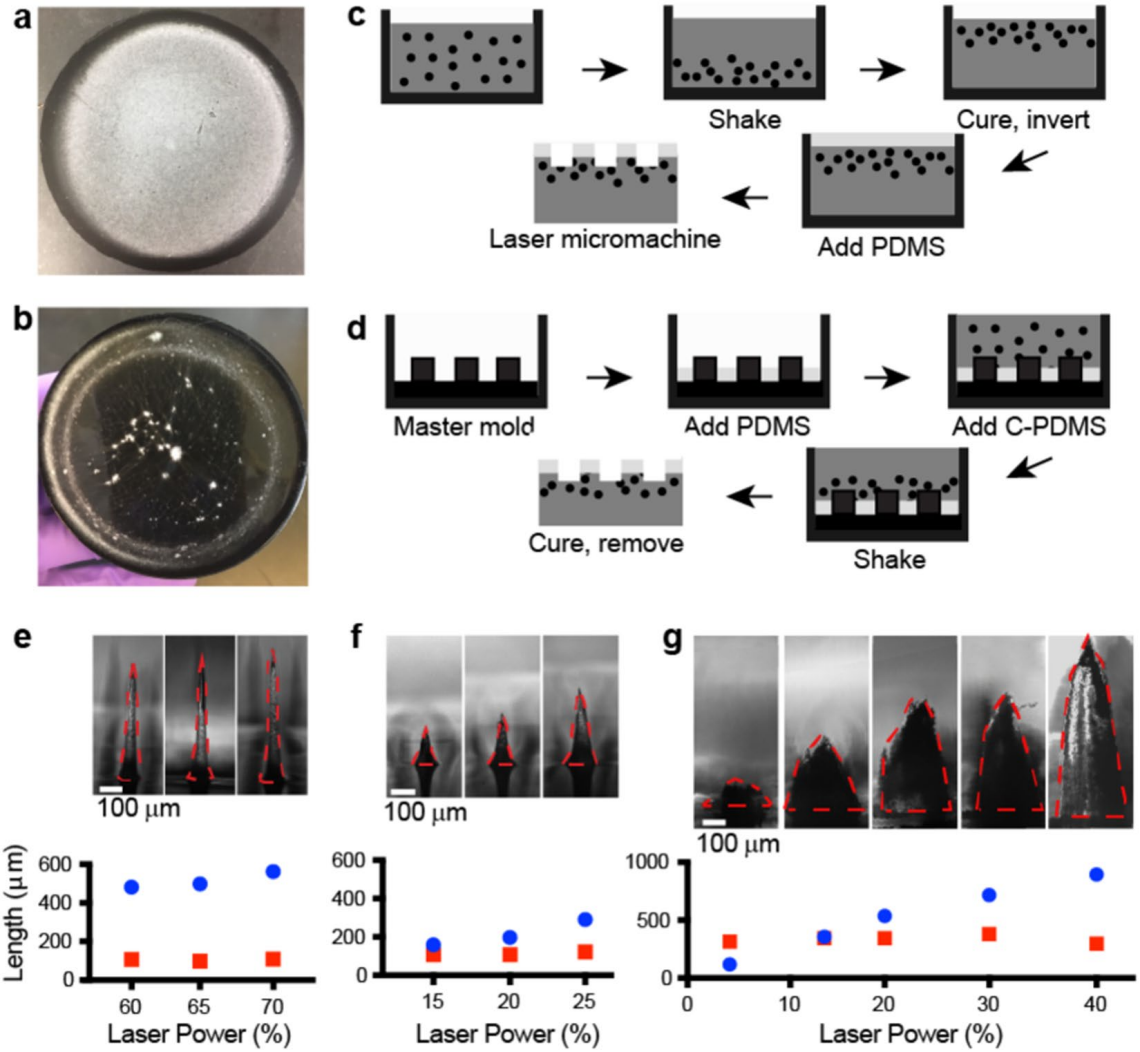
Rapid and inexpensive collector prototyping by subtractive and additive processes using a PDMS-based composite material. (**a**) PVA fibers electrospun onto the side of the cured carbon black-PDMS mixture with concentrated carbon black particles, showing uniform fiber deposition. (**b**) PVA fibers electrospun onto the carbon black-PDMS mixture with diffuse carbon black particles, showing minimal and highly irregular fiber deposition. Schematic of the (**c**) subtractive procedure starting with adding the 7.5 wt% carbon black in PDMS to a mold, then shaking the mixture. This mixture is cured and inverted, then a layer of PDMS is added to the surface and cured. The two-layer material is then patterned using a CO_2_ laser. (**d**)The additive fabrication procedure starts with a master mold. PDMS is added and cured, then C-PDMS is added and the entire mold is placed on a plate shaker. The C-PDMS is then cured, and the completed collector removed from the mold. Representative optical images and measurements of base (□□) and height (●) dimensions of conical features cast from a PDMS collector laser cut with **(e**) a point pattern, (**f**) a circle pattern (diameter: 0.06 mm), or (**g**) a helix pattern (diameter: 0.25 mm, 10 turns). All features used a variable laser power and constant laser speed (95%).

To experimentally evaluate the collector design features predicted to affect fiber deposition in the simulations, we modified the PDMS based materials and collector fabrication process used for our proof-of-concept collectors. In the first iteration of these collectors, the 12.5% w/v C-PDMS was highly viscous, making it difficult to mix and mold the material. To address this challenge, we incorporated carbon black into the PDMS at a lower concentration (7.5 wt%), then poured it into a mold and placed it on a rotating platform shaker to generate turbulence in the mixture and increase the settling velocity of the carbon black particles^35^. The settling of the carbon black was observed qualitatively by electrospinning PVA fibers onto the bottom (Fig. 3a) and top (Fig. 3b) of the C-PDMS after 15 hours of shaking followed by curing. Fiber deposition was uniform across the bottom of the material, likely due to concentration of the carbon black particles (Fig. 3a). In comparison, fiber deposition was sparse and uneven on the top of the material where the particles were likely more dilute (Fig. 3b). Although the shaking step adds time to the collector fabrication process, it enables uniform fiber collection with carbon black concentrations below the 10 wt% threshold needed to achieve conductivity (5*10^−3^ (ohm*cm) ^−1^) in carbon black-PDMS mixtures and below previously reported concentrations for electrospinning collectors (12.5% w/v)^28,34,36^. The small reduction of carbon black concentration reduces the viscosity of the mixture approximately 6-fold, from 148,000 cP to 24,000 cP. This reduction facilitates easy mixing and molding of the material^34^.

We incorporated this modification of the C-PDMS processing into both subtractive and additive fabrication methods. As described previously, the subtractive process starts with preparation and curing of the C-PDMS, followed by addition of PDMS. This completed two-layer material is then micromachined using a CO_2_ laser system (Fig. 3c). While the subtractive process used in the proof-of-concept collectors was well suited to relatively shallow patterns (feature depth less than 1 mm), fabrication of deeper patterns required multiple passes of the laser. Therefore, we evaluated an additive process that is better suited to larger patterns. For the additive process (Fig. 3d), an appropriate volume of PDMS is first added to a master mold to achieve the desired thickness and cured. Next, the C-PDMS mixture is added to the mold, shaken, and cured. For the experimental studies used to verify the simulation results, we chose to use the subtractive fabrication method to create conical features in the two-layer PDMS-based material with diameters ranging from 98 to 378 μm and heights ranging from 159 to 895 μm (Fig. 3e–g).

From start to finish, both collector fabrication processes require approximately 30 minutes of active time over 2 days. The entire collector fabrication process is completed with inexpensive materials and laboratory equipment that is readily available. While potential alternative collector materials systems such as silicon have higher stiffness and higher conductivity, this PDMS based system is favorable for proof of concept studies, rapid collector design iteration, or for settings where a cleanroom for silicon processing is not available.

### Electrospun fiber conformation to 3D collector pattern depends on insulative layer thickness and feature height

**Figure 4:**
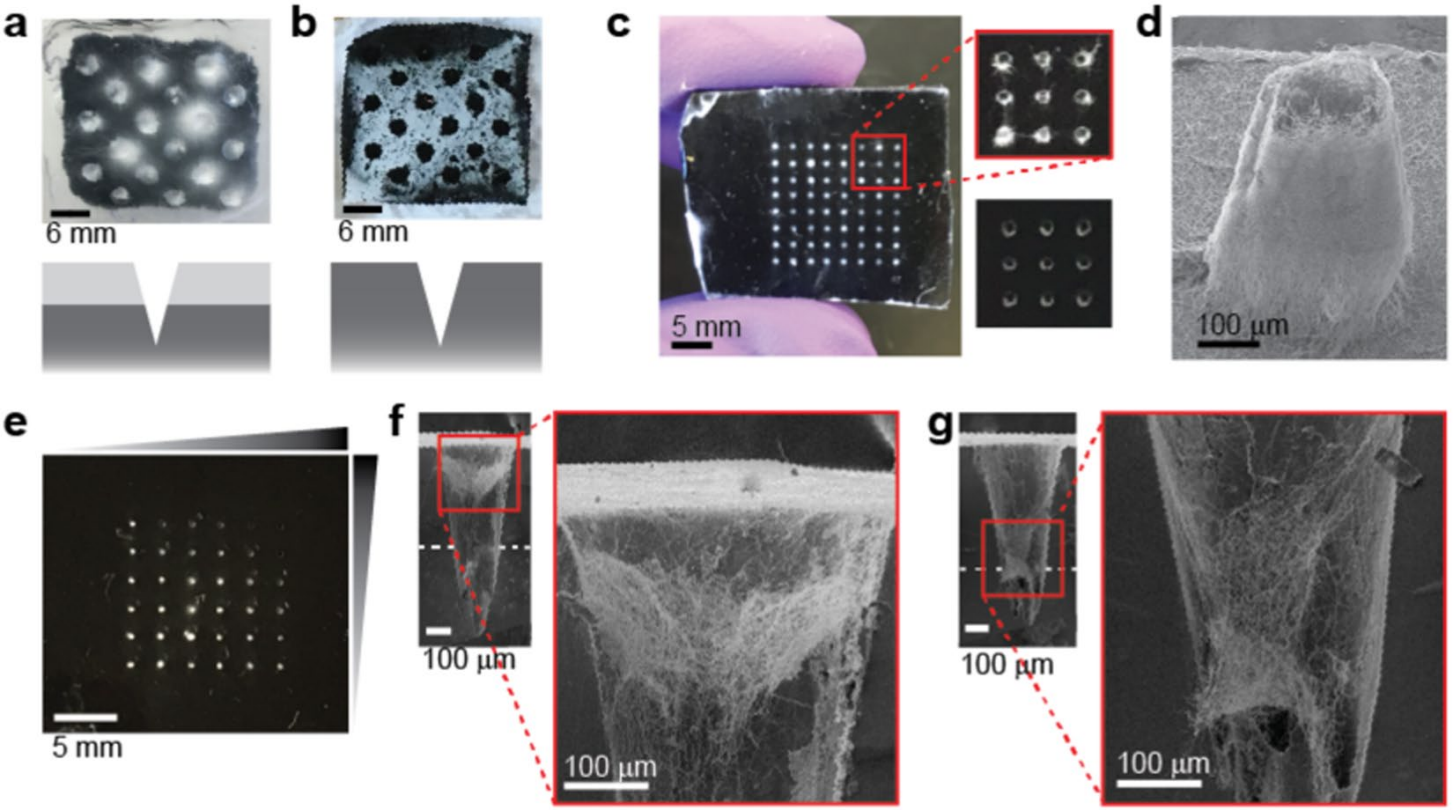
Electrospun fiber conformation to 3D collector pattern depends on insulative layer thickness and feature height. PVA fiber collection on millimeter-scale PDMS-based patterned collectors **(a**) with and (**b**) without a 600 μm thick PDMS surface layer demonstrates the need for the insulative layer to achieve fiber deposition in the micropatterns. Conical patterns had diameter 3 mm, 3.25 mm height, and 5.5 mm spacing. (**c**) Micro-scale PDMS-based collector with (top inset) and without (bottom inset) PVA fiber deposition. Collector had 400 μm PDMS thickness and conical patterns with 269 ± 5 μm diameter, 522 ± 6 μm height, 1400 μm spacing. (**d**) SEM image of fibers carefully removed from micro-scale collector demonstrates fiber patterning in three dimensions. (**e**) Top-down view of micropatterned collector with gradient PDMS layer thickness containing PVA electrospun fibers. This collector contained conical patterns with 364 ± 16 μm diameter, 777 ± 20 μm height, 1600 μm spacing, and a PDMS layer that ranged from 400 to 580 μm. Fiber deposition is less visible in areas of the collector with thicker PDMS layer. (**f**) Cross-sectional SEM image of the gradient PDMS thickness micropatterned collector at a region with 400 μm PDMS thickness and (**g**) a region with 500 μm PDMS thickness containing PVA electrospun fibers. The approximate location of the insulative layer is denoted by the white dashed line. These SEM images were processed in Adobe Photoshop to improve visibility of fibers. Contrast was set to 100 and exposure offset was set to −0.1. For all electrospinning experiments, the needle-collector separation distance was 10 cm. The solution flow rate varied between 1.5–5 μL/min and the applied voltage varied between 7.5–8.5 kV because of the differences in solution properties. For millimeter scale collectors, fibers were electrospun for approximately 5 minutes, and for microscale collectors, fibers were electrospun for 1–2 minutes.

After identifying key factors predicted to affect fiber deposition patterns and developing a strategy for rapidly prototyping different collector patterns, we set out to experimentally validate our simulation results. The first goal of the experimental evaluation was to assess the effect of the insulative surface layer compared to a fully conductive patterned collector. An additive strategy was used to generate millimeter-scale patterns (3 mm diameter, 3.25 mm height, and 5.5 mm spacing) in a collector with and without a 600-μm thick insulative PDMS layer (Fig. 4a,b). PVA fibers deposited only on the surface of the fully conductive collector without the insulative layer, but fibers deposited densely in the patterns on the insulated two-layer collector (Fig. 4a,b). These results are in agreement with the simulations, which calculated a 365 V/m higher ΔE for the collector with the insulative PDMS layer. These studies experimentally demonstrate the function of an insulative surface layer to achieve fiber deposition within recessed patterns.

The PDMS-based collectors were then scaled down by approximately 10-fold to verify that the patterning effect was valid at smaller length scales (conical pattern: 269 ± 5 μm diameter, 522 ± 6 μm height, 1400 μm spacing, 400 μm insulative PDMS layer thickness). Based on the simulations (ΔE = 882 V/m), we expected to observe fiber deposition in the patterns for this collector. We observed a similar patterning effect for this microscale collector, with fibers deposited densely within the patterns in 1-2 minutes and little to no deposition on the collector surface (Fig. 4c). To verify that we were not simply observing fibers bridging over the patterns, we increased the electrospinning time to create a fiber mat strong enough to be removed from the collector. We then imaged the fibers with scanning electron microscopy and observed that the fibers conformed to the patterned collector in three dimensions (Fig. 4d). Coupled with the results for the millimeter-scale collector, these results confirm that with appropriate selection of feature height and insulative layer thickness, the fiber patterning strategy is feasible on a range of length scales.

To better understand the effect of insulative layer thickness on fiber deposition, we fabricated a microscale collector with a gradient insulative PDMS layer thickness and constant feature geometry, dimensions, and spacing. This collector contained conical patterns (364 ± 16 μm diameter, 777 ± 20 μm height, 1600 μm spacing), and an insulative PDMS layer that ranged from 400 to 580 μm. Our simulations calculated that the ΔE ranged from 647 V/m for the 400 μm PDMS thickness to 705 V/m for the 580 μm PDMS thickness. PVA fibers were deposited densely in the patterns over a majority of the collector in 1-2 minutes, with more visible fibers in the patterns with a thinner insulative PDMS layer and fewer visible fibers visible in patterns with a thicker insulative PDMS layer (Fig. 4e). When cross-sections of this fiber-containing collector were inspected by SEM, we observed that the fiber deposition depth was modulated by the insulative PDMS thickness. Fibers deposited near the openings of the patterns with a 400-μm thick PDMS layer (Fig. 4f), while fibers appeared to be deposited toward the bottom of the pattern for a 500 μm-thick PDMS layer (Fig. 4g). The deeper fiber deposition in patterns with a thicker insulative PDMS layer is possibly related to the increased ΔE compared to patterns with a thinner insulative PDMS layer.

Overall, the experimental evaluation of our two-layer collectors for *in situ* fiber patterning agreed with the predictions of the finite element method simulations. Electrospun fibers deposited into patterns with a range of feature heights from 3.25 mm to 522 μm through appropriate selection of insulative layer thickness, corroborating a key finding from the simulations. Because the simulations do not capture some key factors related to fiber material and electrospinning dynamics^37,38^, they cannot be used alone to precisely determine fiber deposition patterns. However, agreement in trends between the experiments and the simulations suggests that the simulation results can be used as a guide to direct the design of two-layer collectors for three-dimensional patterning of electrospun fibers.

### *In situ* patterning strategy shows versatility for physicochemically diverse polymers

**Figure 5:**
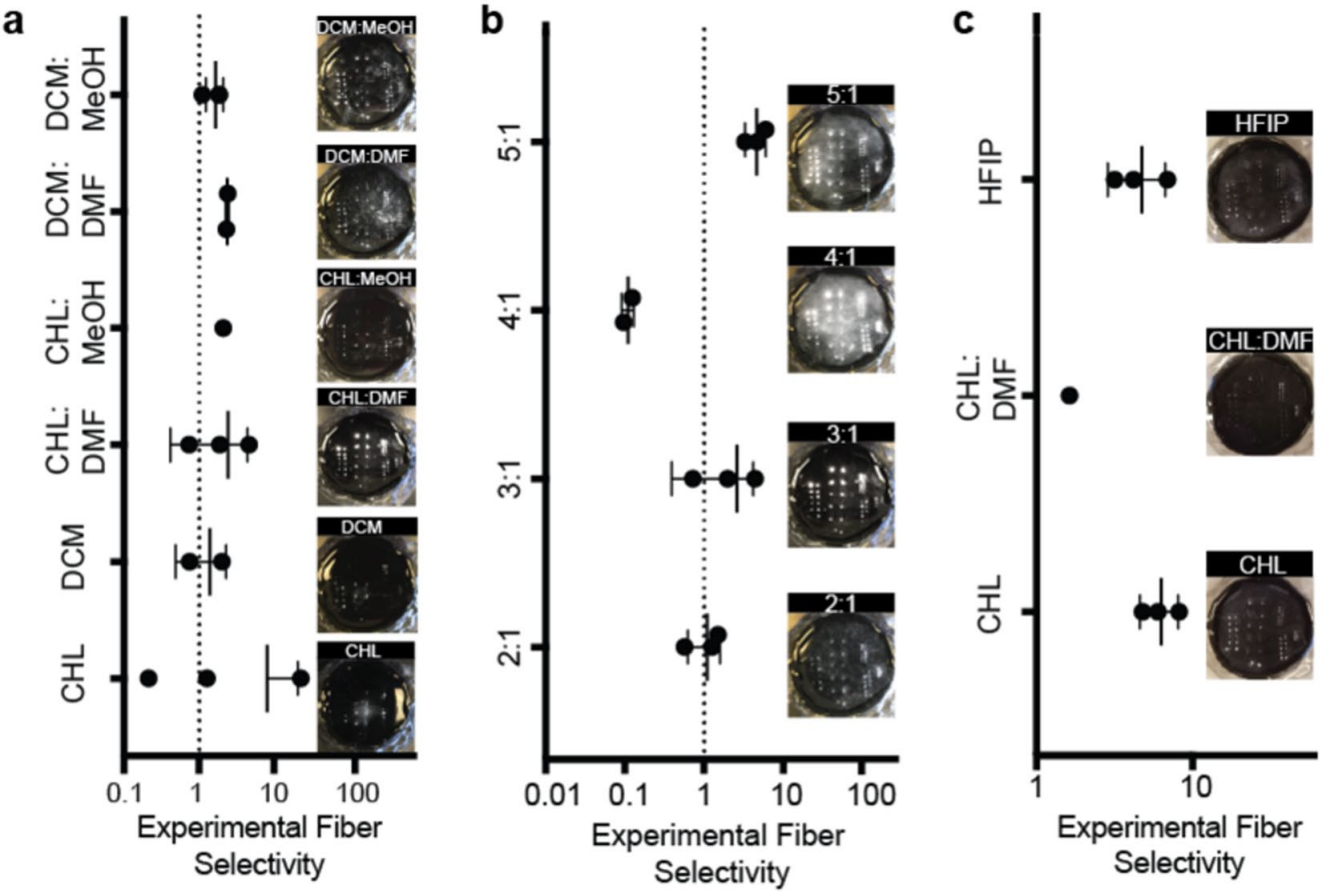
*In situ* patterning strategy shows versatility for physicochemically diverse polymers. Ratio of fiber mass in the micropatterns to fiber mass on the collector surface and representative top-down images of fibers on micropatterned collectors for (**a**) PCL fibers electrospun from different solvents, (**b**) PCL fibers electrospun from different CHL:DMF ratios, (**c**) PLGA fibers electrospun from different solvents. (n = 3, error bars represent standard deviations). The same collector with conical micropatterns of base diameter 364 ± 16 μm, height 777 ± 20 μm, spacing ranging from 0.5 mm to 4 mm, and insulative layer thickness 0.4 mm was used for all electrospinning experiments. PCL fibers were electrospun at a voltage of 17 kV, while PLGA fibers were electrospun at a voltage of 18 kV. All fiber samples were electrospun at a 5 μL/min flow rate and 25 cm tip to collector distance for 2 minutes.

Another desirable feature of our patterning strategy is its ability to accommodate *in situ* electrospinning of a variety of fiber materials with different physicochemical properties. We evaluated this experimentally since the properties of the fibers and their behavior in the electric field were too complex to simulate accurately. We chose to demonstrate patterning of polycaprolactone (PCL) and poly(lactic-co-glycolic acid) (PLGA) here because they are widely used in drug delivery and tissue engineering, and organic solutions of these polymers have very different properties than the aqueous PVA solution used in our original experimental validation studies^4,12,14,24^. Because the PDMS-based collectors are much less conductive than standard metal collectors used for electrospinning, the low polarity polyester solutions required experimentation to identify formulations with reproducible results, high yield, and selectivity for the pattern. For each polyester, we measured solution properties and fiber output from electrospinning (2 minutes) onto a collector containing five 2 × 6 arrays of conical patterns (364 ± 16 μm diameter, 777 ± 20 μm height, 500–4000 μm spacing, 500 μm insulative layer thickness). We also calculated the yield and the experimental fiber selectivity (S_E_), defined here as the ratio of fiber mass deposited in the patterns to fiber mass deposited on the collector surface (Table 2, Fig. 5a).

**Table 2:** Polymer solution properties for patterned electrospun fibers.

	Solvent	Conductivity (μS/cm)	Viscosity (Pa*s)	Output (mg)^a, b^	Yield (%)^a^	S_E_
PCL Solvent Selection^c^	CHL	0.00	6.66	0.55 ± 0.32	45.8 ± 26.6	8.09
	DCM	0.00	3.45	0.1 ± 0.01	8.3 ± 0.8	1.39
	DCM:DMF	0.59	5.54	0.35 ± 0.25	29.1 ± 20.8	2.38
	DCM:MeOH	0.81	2.64	0.14 ± 0.07	11.6 ± 5.8	1.67
	CHL:MeOH	0.56	2.58	0.07 ± 0.04	5.8 ± 3.3	2.11
	CHL:DMF	0.24	4.56	0.83 ± 0.39	69.1 ± 32.5	2.43
PCL Conductivity Evaluation^c^	2:1 CHL:DMF	0.22	3.91	0.57 ± 0.32	47.5 ± 26.6	1.09
	4:1 CHL:DMF	0.20	5.33	0.76 ± 0.26	63.3 ± 21.6	0.10
	5:1 CHL:DMF	0.17	5.36	1.21 ± 0.49	100.8 ± 40.8	4.90
PLGA Solvent Selection^d^	CHL	0.00	2.03	1.11 ± 0.16	111 ± 16	6.71
	CHL:DMF	0.36	3.72	0.17 ± 0.08	17 ± 8	2.06
	HFIP	0.09	10.84	1.04 ± 0.40	104 ± 40	5.21

^a^Mean ± standard deviation of n = 3 electrospinning replicates, ^b^Output is reported as total fiber mass on the collector after 2 minutes of electrospinning, ^c^All PCL solutions were prepared at 12 wt%, ^d^All PLGA solutions were prepared at 10 wt%.

To improve the *in situ* patterning of PCL fibers, we first prepared PCL solutions at 12 wt% in various solvents and solvent mixtures with different conductivity and viscosity, factors that are known to affect fiber morphology and deposition patterns^25,27,37,38^. In general, higher fiber yield was observed for PCL solutions with higher viscosity and lower conductivity (chloroform (CHL), 3:1 chloroform: dimethylformamide (CHL: DMF)) (Table 2). The CHL: DMF mixture was prioritized for further studies because of its high S_E_ combined with its high yield and uniform deposition across the collector (Fig. 5a). In the next iteration of PCL solution development, we tuned the solution conductivity by changing the ratio of CHL:DMF rather than through addition of salts because of the poor yield and S_E_ of the highest conductivity solutions in the initial solvent evaluation (Table 2). The best yield (100.8%) and S_E_ (4.9) were observed for the PCL solution in 5:1 CHL: DMF, which had approximately 30% lower conductivity and approximately 20% higher viscosity than the starting 3:1 CHL:DMF solution (Fig. 5b). While the yield of this solution still varied between replicates, S_E_ was reproducible. Ultimately, a 12 wt% solution of PCL in 5:1 CHL: DMF was identified to be best suited for our *in situ* patterning, producing a nearly 20-fold increase in fiber yield and nearly 50-fold increase in S_E_ compared to the worst performing solution. A similar approach was used to identify a formulation with high yield and S_E_ for PLGA fibers. Here, we found that a 10% PLGA solution in CHL provided the best yield (111%) and S_E_ (6.71) (Fig. 5c).

Performance of PLGA solutions followed similar trends to the PCL solutions, with higher viscosity and lower conductivity solutions producing the highest S_E_ and yield (Table 2). This effect is potentially caused by the increased net charge density of electrospun fibers produced from higher conductivity solutions^39^. These fibers may not completely discharge after deposition on the collector, leading to repulsion of incoming fibers^26,40^. Therefore, future studies attempting to pattern fibers containing high concentrations of conductive agents could encounter reduced fiber yield or compromised fiber pattern quality. However, these studies could explore the use of AC potentials or a modified collector material to reduce surface charge accumulation and overcome this challenge^26,40^. Overall, the polymer solution development performed here provides a framework for adapting virtually any fiber formulation containing active agents, other additives, or different fiber materials for use with our patterning strategy.

### Fabrication of mechanically robust integrated fiber microneedles

**Figure 6:**
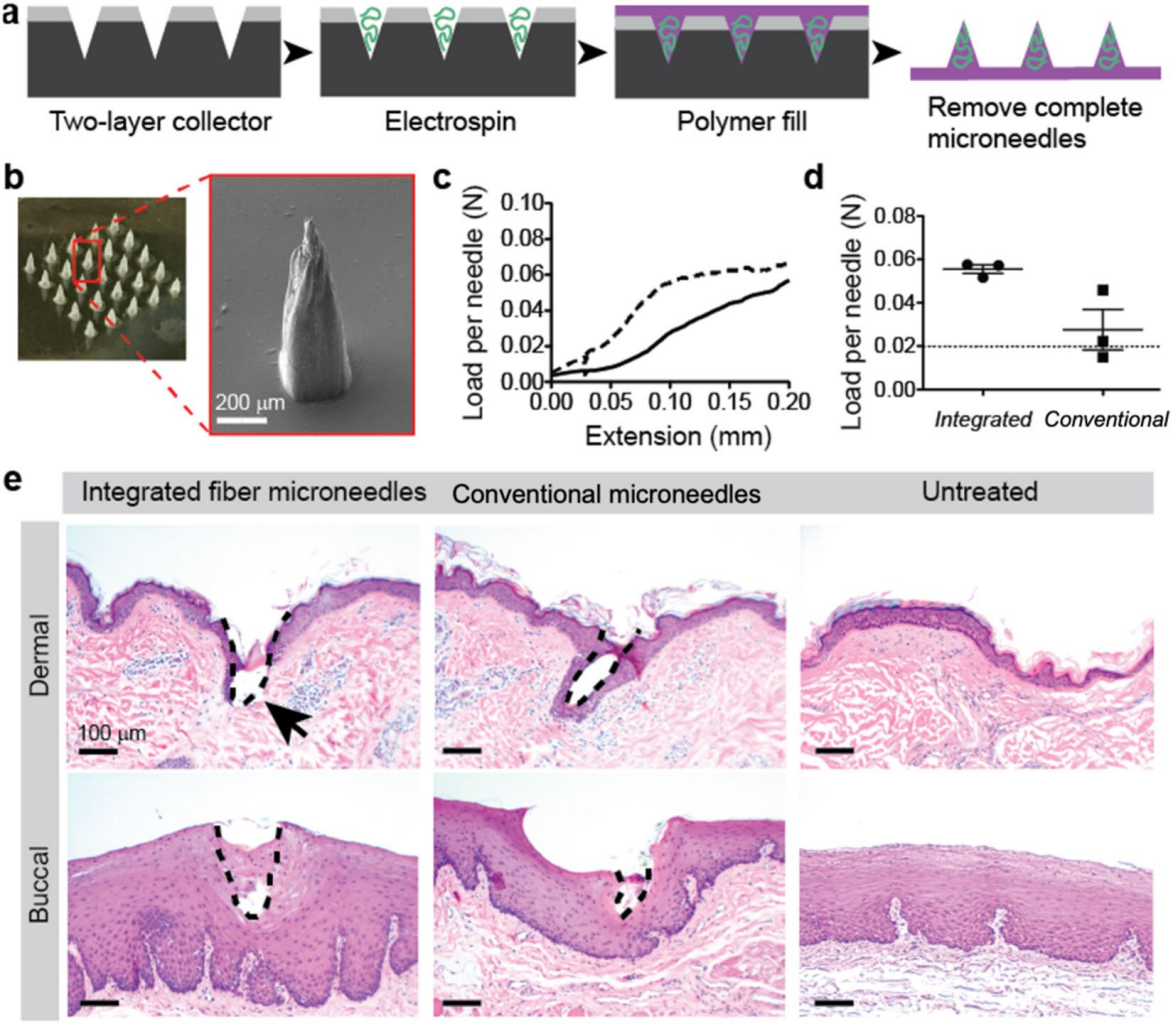
Fabrication of mechanically robust integrated fiber microneedles. (**a**) Integrated fiber microneedle fabrication approach. (**b**) Optical microscope image of a completed integrated fiber microneedle array and (inset) SEM image of a single integrated fiber microneedle. (**c**) Graph of load and extension per needle for compression of 3 × 3 microneedle arrays between two steel plates using an Instron universal testing system. Curve represents the mean of three integrated fiber microneedle arrays (dashed line) and conventional matrix microneedles (solid line) from separate PDMS-based collectors. Error bars omitted for graph clarity. (**d**) Failure forces per needle for integrated fiber microneedles compared to conventional matrix microneedles. For all samples measured with this method, the failure force was taken as the load at 0.1 mm extension (n = 3, error bars represent standard deviations). (**e**) Optical microscopy of dermal and buccal tissue treated with conventional matrix microneedles or integrated fiber microneedles demonstrating integrated fiber microneedle disruption of the dermal stratum corneum and viable epidermis and conventional matrix microneedle and integrated fiber microneedle access to the epithelium of buccal tissue. Untreated dermal and buccal tissue controls are provided to demonstrate the native structure of both tissues.

As a demonstration of one application of this fiber patterning strategy, we used it to integrate electrospun fibers with dissolving microneedles. Here, we generated a two-layer collector in the shape of a microneedle array, deposited electrospun fibers within the collector patterns, and filled the collectors with a polymer solution to create integrated fiber microneedles (Fig. 6a). The integrated fiber microneedles used for these studies consisted of PCL fibers electrospun from the optimized solution described in the previous section onto a collector with conical patterns (364 ± 16 μm diameter, 777 ± 20 μm height, 1000 μm spacing, 500 μm insulative layer thickness). After electrospinning for 2 minutes, an aqueous solution of poly(acrylic acid) was applied to the collector to create a dissolvable matrix around the electrospun fibers and a backing layer (Fig. 6b)^41–43^. In a cross-sectional image of the completed integrated fiber microneedle device, we observed an interconnected network of electrospun fibers dispersed throughout the polymer matrix material along the entire length of the needle (Figure S1). This data suggests that the 3D-pattern of the electrospun fibers is maintained after the addition of polyacrylic acid. Based on previous studies of polyacrylic acid microneedles, we anticipate that the matrix material will dissolve rapidly on contact with the interstitial fluid in the skin to deliver a 3D network of electrospun fibers^41–43^.

Because the primary function of microneedles is their ability to puncture tissue^44^, mechanical characterization of the integrated fiber microneedles was evaluated. Compressive testing of 3 × 3 microneedle arrays indicated that integrated fiber microneedles were stiffer than microneedles without fibers fabricated by the same process (Fig. 6c). Integrated fiber microneedle failure forces exceeded previously established targets for tissue puncture^45^, with an average failure force per needle of 0.055 N (Fig. 6d). In contrast, conventional matrix microneedles without fibers failed at a lower force of 0.027 N/needle. This observation could be related to the microarchitecture of the integrated fiber microneedle device (Figure S1). In previous studies, a similar microarchitecture enabled efficient transfer of compressive force from the matrix material to the fiber reinforcement material, resulting in improved compressive mechanical properties^46,47^. The failure force of the integrated fiber microneedles was reproducible, suggesting a consistent fabrication process. Since the predicted force per needle for tissue insertion is approximately 0.02 N, these needles have a factor of safety of nearly 3, meaning that they should reliably and completely puncture tissue^44^.

To validate that the integrated fiber microneedle patch could effectively puncture tissue, we applied the patch to non-human primate dermal and buccal tissue *ex vivo*. These tissues are of interest for drug and vaccine delivery and possess different mechanical properties^48,49^. The results indicate that only integrated fiber microneedles penetrated both the stratum corneum and the viable epidermis of dermal tissue (Fig. 6e). In buccal tissue, the integrated fiber microneedles penetrated to a depth of approximately 240 μm, 30% of the needle height. Conventional matrix microneedles without fibers penetrated approximately 130 μm in the buccal tissue, potentially because they are mechanically weaker than the integrated fiber microneedles. In previous studies, penetration depths of 30-50% of the microneedle height have been sufficient to achieve both bolus and sustained delivery with approximately 30% delivery efficiency^50,51^. As such, we expect to see comparable delivery with a 30% penetration depth of our microneedles in buccal tissue. Future studies applying integrated fiber microneedles for specific drug delivery applications could alter microneedle dimensions or application force to achieve the desired penetration depth^52,53^.

Integrated fiber microneedles could provide an alternative delivery modality for electrospun fibers, and they could expand the functionality of existing microneedles by enabling stable drug formulation and release from the fiber scaffolds (Figure S2). Dapivirine loaded fibers were deposited onto the two-layer collector with 71% yield and an experimental selectivity of 1.35 (Table S1). Conventional matrix microneedles without fibers loaded at 30 wt% dapivirine resulted in Ostwald ripening of the drug into large particles and needle deformation (Figure S2c-e). Because these microneedles were not suitable for further evaluation, integrated fiber microneedles and conventional matrix microneedles were prepared at a lower 15 wt% dapivirine loading for use in release studies. After an initial burst of approximately 5%, dapivirine release from the integrated fiber microneedles was linear over time with an approximate rate of 0.1% per day and 0.2% per day for 15 wt% and 30 wt% dapivirine loading, respectively. In contrast, the control group of conventional matrix microneedles with 15 wt% dapivirine loading showed negligible release before one week (Figure S2f). We observed during the release experiment that the drug loaded conventional matrix microneedles became white and opaque compared to a control group of conventional matrix microneedles without drug (Figure S2g-h). The observed opacity is likely caused by drug crystallization in the release conditions, which could also explain the slow release rate^54^. Future studies could further explore the drug delivery capabilities of the integrated fiber microneedle system, including its ability to achieve localized, tunable release of bioactive agents *in vivo*. This example application illustrates how this fiber patterning strategy could enable the development of new devices with valuable attributes for future biomedical applications.

## Materials and Methods

### COMSOL simulation setup and parameters

The effect of various two-layer patterned collector designs on the electric field near the collector was evaluated using finite element method simulations (COMSOL Multiphysics version 5.1). The 2D schematic of the collector and the electrospinning setup for each simulation was prepared in AutoCAD (Autodesk AutoCAD 2015). For every simulation, the tip to collector distance was set to 10 cm. The geometry included a boundary around the entire setup. The materials assigned to each geometry in the simulation matched the materials used in experimental evaluations. Charge conservation was maintained in the simulation, and the zero-charge boundary condition was assigned to the outer boundary of the geometry. The initial value for electric potential was set to zero across the entire geometry. The applied voltage of 7.5 kV was assigned to the geometry representing the needle in the electrospinning setup. This is an actual voltage that was used for the experimental evaluation. The geometry representing the collector was grounded. Geometries were rendered with an extra fine physics-controlled mesh. The electric field at different points relative to the collector was calculated using the potential computed by the simulation.

### Statistical analysis of simulations

All simulation experimental design and statistical analysis was performed using Design Expert software (version 9.0.4, Stat-Ease Inc.). The central composite design included factorial points matched to the full factorial design, 5 center points, and 9 star points (α = 2.5). Results from both experimental designs were analyzed using ANOVA and the F-test. The results of the central composite design were used to construct a quadratic model (R^2^ = 0.9488) that can be used to predict ΔE for any given set of control factor inputs (Eq. 1).

1 $$\begin{array}{rcl}\Delta E & = & 1286+(293\,\ast \,thickness)-(35\,\ast \,spacing)+(31\,\ast \,width)-(963\,\ast \,height)\\  & + & (106\,\ast \,thickness\,\ast \,height)-(53\,\ast \,thicknes{s}^{2})+(0.382\,\ast \,heigh{t}^{2})\end{array}$$

Results from the factorial design were tested for normality using the Shapiro-Wilk test, which resulted in a W-value of 0.967 and a p-value of 0.561, indicating that terms not selected for the model were normally distributed. The central composite design results were tested for normality by inspecting the normal probability plot for linearity.

### Design and preparation of two-layer patterned collectors

To prepare the millimeter-scale two-layer patterned collectors, first an array of cones was designed in Autodesk Inventor and 3D printed in poly(lactic acid) with a FlashForge Finder at the University of Washington CoMotion Makerspace. A layer of PDMS (mixed at 1:10 ratio curing agent: pre-polymer, Sylgard 184, Dow Corning) was added to the array and cured at room temperature for 24 hours. C-PDMS was prepared by first incorporating 7.5 wt% carbon black (Vulcan XC 72 R, particle size 50 nm, Fuel Cell Store) into the PDMS pre-polymer using an overhead stirrer (IKA Eurostar) with a propeller attachment at 250 rpm. When all the carbon black was incorporated, the PDMS curing agent (1:10 ratio to the pre-polymer) was added and mixed for 5 minutes at 250 rpm. The completed C-PDMS mixture was added to the master cone array until the cone tips were completely covered. The entire mold was then placed on a rotating platform shaker at the highest setting for 15 hours. The C-PDMS was then cured in an oven at 37 °C for 24 hours, and the completed collector was removed from the master mold. To prepare the micro-scale two-layer patterned collectors, first the complete C-PDMS mixture was added to a 40 mm square plastic weigh boat, placed on a rotating platform shaker at the highest setting for 15 hours, and cured in an oven at 37 °C for 24 hours. The C-PDMS material was then inverted, and PDMS (0.4 mL) was added to the surface. After this two-layer material was fabricated, the conical patterns were created in the material using a VLS 3.60 CO_2_ laser system (Universal Laser Systems) in vector mode with the enhance feature selected. To create the 364-μm diameter base and 777-μm height conical patterns used for the microneedle application, a helix pattern with 10 turns and a diameter of 0.25 mm was plotted using the laser cutter. Laser power was set to 40% and speed was set to 90%.

### Rheology

Viscosity of carbon black-PDMS mixtures was measured using a TA-Instruments Rheometer (Model ARG2) with a 40 mm diameter, 2° steel cone geometry. Data were collected in frequency sweep oscillation mode with constant small strain of 4%. Viscosity was calculated by dividing G” by the angular frequency. Viscosity values are reported at an angular frequency of 1 rad/s.

### Polymer solution preparation and electrospinning

Solutions of polyester materials were prepared by combining PCL (Sigma Aldrich, average M_n_ 80,000) or PLGA (Lactel Absorbable Polymers, 50:50 L:G ester terminated, 0.55-0.75 dL/g inherent viscosity in HFIP) in appropriate solvents, followed by stirring overnight on a rotisserie style shaker at room temperature. All PCL solutions were prepared at 12 wt%, and all PLGA solutions were prepared at 10 wt%. Solutions of PVA (Spectrum Chemical, M_w_~105 kDa, P1180) were prepared in water at 10 wt% by stirring overnight with gentle heating. Polymer solution conductivity was determined using a conductivity meter (Thermo Scientific Orion Star A212) and viscosity was measured on a rheometer (TA Instruments AR-G2). Polymer solutions were loaded in a glass syringe fitted with a 22 G blunt tipped needle. The polymer solution was dispensed from the syringe using a syringe pump (New Era Pump Systems, Inc.) at a 2 μL/min flow rate. The patterned collector of interest was fixed to a custom holder that inserted copper wires to the back of the collector. The wires were pierced through a polyethylene foam block to hold the collector at the proper height and to prevent fiber deposition on objects other than the collector. The ground from the power source (Gamma High Voltage Research) was attached to these wires and the positive lead was attached to the base of the needle. The applied voltage varied between 7.5 and 18 kV depending on the polymer solution and the tip to collector distance. Fiber samples were electrospun for 1–5 minutes.

### Microscopy

All optical microscope images were obtained with a Nikon Eclipse Ti microscope with a Nikon Digital Sight DS-Fi2 camera. After fiber deposition on the patterned collectors, fibers were carefully removed with clear packaging tape and mounted on glass microscope slides for imaging. Images were captured at 20X magnification. The same contrast and brightness settings were used for all images to enable unbiased threshold analysis. SEM imaging of electrospun fibers on the two-layer patterned collectors, patterned fibers alone, and completed microneedles was performed on a JEOL JSM7400F cold field emission scanning electron microscope. All samples were coated with a 3 nm layer of gold-palladium prior to SEM imaging.

### Image analysis and calculation of experimental fiber selectivity

All image analysis was performed in ImageJ. The percent area of the collector surface covered with fibers within the microscope image field was determined by converting an optical microscope image to 8-bit, applying an auto-threshold to make all fibers appear completely black, and measuring percent area. This calculation was repeated for images from four different locations on the collector surface, and an average percent area was calculated. Fiber diameters were measured from at least 50 different fibers in SEM images. The percent area from the threshold analysis and the fiber diameter were used to estimate the volume of fibers covering the collector surface. The volume value was then multiplied by a scaling factor to estimate the fiber volume across the entire collector surface. Assuming the density of the fibers was equal to reported density of the raw polymer material, we calculated the mass of fibers on the collector surface. The mass of fibers in the patterns was then determined by subtracting the calculated mass of fibers on the collector surface from the measured total fiber mass on the collector. Finally, the ratio of fiber mass in the patterns to fiber mass on the collector surface was calculated.

### Microneedle fabrication

After electrospinning, the microneedle mold two-layer collectors were filled with an aqueous PAA solution (8.75 wt%) by centrifugation at 1000x g for 1 hour. Excess PAA was removed from the collector surface and a higher concentration PAA solution (35 wt%) was added to the collector to create a strong backing layer. Microneedles were dried at room temperature for 2 days, and then the complete integrated fiber microneedle patch was gently removed from the collector. Once the collector is fabricated, the integrated fiber microneedle patches can be fabricated with an active time of approximately 1 hour over two days.

### Microneedle mechanical characterization

Compression testing of microneedle arrays was performed on an Instron Universal Testing System (Model 5943). An array size of 3 × 3 was used for all groups and replicates. The microneedle array was mounted to a microscope slide with double-sided tape, and the slide was then secured to the fixed base plate with double-sided tape. A flat stainless-steel disc adapter was attached to the load cell. The load cell moved toward the fixed base plate at a rate of 20 μm/s. The instrument began collecting data when the measured load exceeded 0.08 N. The experiment ended either when the load reached 25 N or when the length of the experiment reached 45 seconds.

### Tissue puncture and histology

Rhesus macaque buccal and dermal tissue was obtained from the Washington National Primate Research Center tissue donor program. Freshly excised buccal tissue was briefly dried with a Kimwipe, then either conventional matrix microneedles or integrated fiber microneedles were applied to the tissue using forceps for 30 seconds, followed by incubation at 37 °C for 5 minutes to allow for microneedle patch dissolution. Dermal tissue from the arm was shaved before harvesting and treated in the same way. Tissues were rinsed with PBS, and then fixed in formalin overnight. Tissue processing and staining was performed by the Pathology Research Services Laboratory at the University of Washington School of Medicine Department of Pathology. Fixed tissues were embedded in paraffin, and 5 μm thick sections were obtained using a microtome. Tissue microarchitecture was visualized using hematoxylin and eosin staining.

## Supplementary information

Supplementary Materials.

## Data Availability

The datasets generated during and/or analyzed during the current study are available from the corresponding author on reasonable request.
