# Author Correction: *In situ* 3D-patterning of electrospun fibers using two-layer composite materials

**DOI:** 10.1038/s41598-020-70777-6

**Published:** 2020-08-17

**Authors:** R. L. Creighton, J. Phan, K. A. Woodrow

**Affiliations:** Department of Bioengineering, University of Washington, Seattle, WA 98195 USA

Correction to: *Scientific Reports* https://doi.org/10.1038/s41598-020-64846-z, published online 14 May 2020

The original Article contained errors which have now been corrected.

The authors identified an error which was found in the central composite design of experiments. The potential measurement and resulting ∆E measurement for a run testing one of the boundary conditions for feature height (height = 0.01 mm) was incorrect. Fixing this error did not significantly change the mean ∆E value for all of the runs (p-value = 0.8080). When a new model was fit to the corrected data, changes were observed in the calculated intercept and several of the equation coefficients. The new model resulted in a reduction in the predicted ∆E for feature heights less than 3 mm (average difference in ∆E =  − 722 V/m) and feature heights greater than 8 mm (average difference in ∆E =  − 243 V/m), and an increase in ∆E for feature heights between 4 mm and 8 mm (average difference in ∆E = 130 V/m) (Figure 1). The change in predicted ∆E was only statistically significant (p-value < 0.05) for feature heights less than 3 mm.

**Figure 1:**
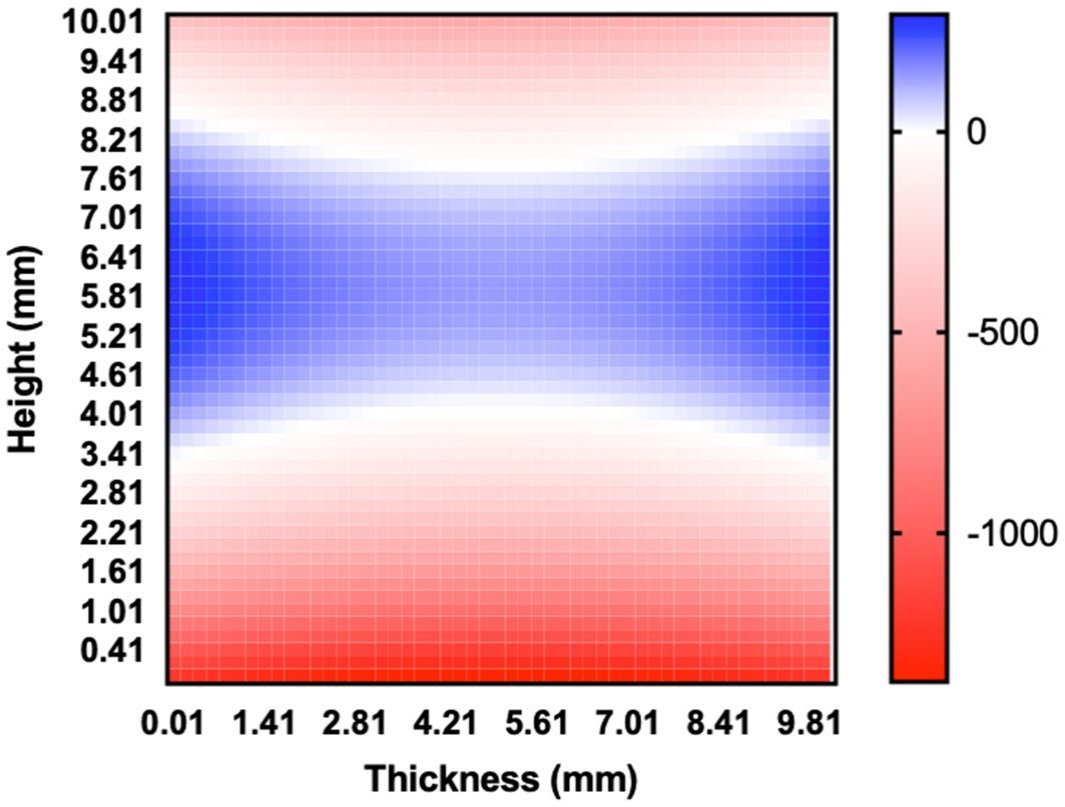
.

Correction of this error affects Table 1 and Figure 2, the correct versions of which now appear in the PDF and HTML versions of the Article. The original, incorrect version of Figure 2 appears below as Figure 2. The original, incorrect version of Table 1 appears below as Table 1.

**Figure 2:**
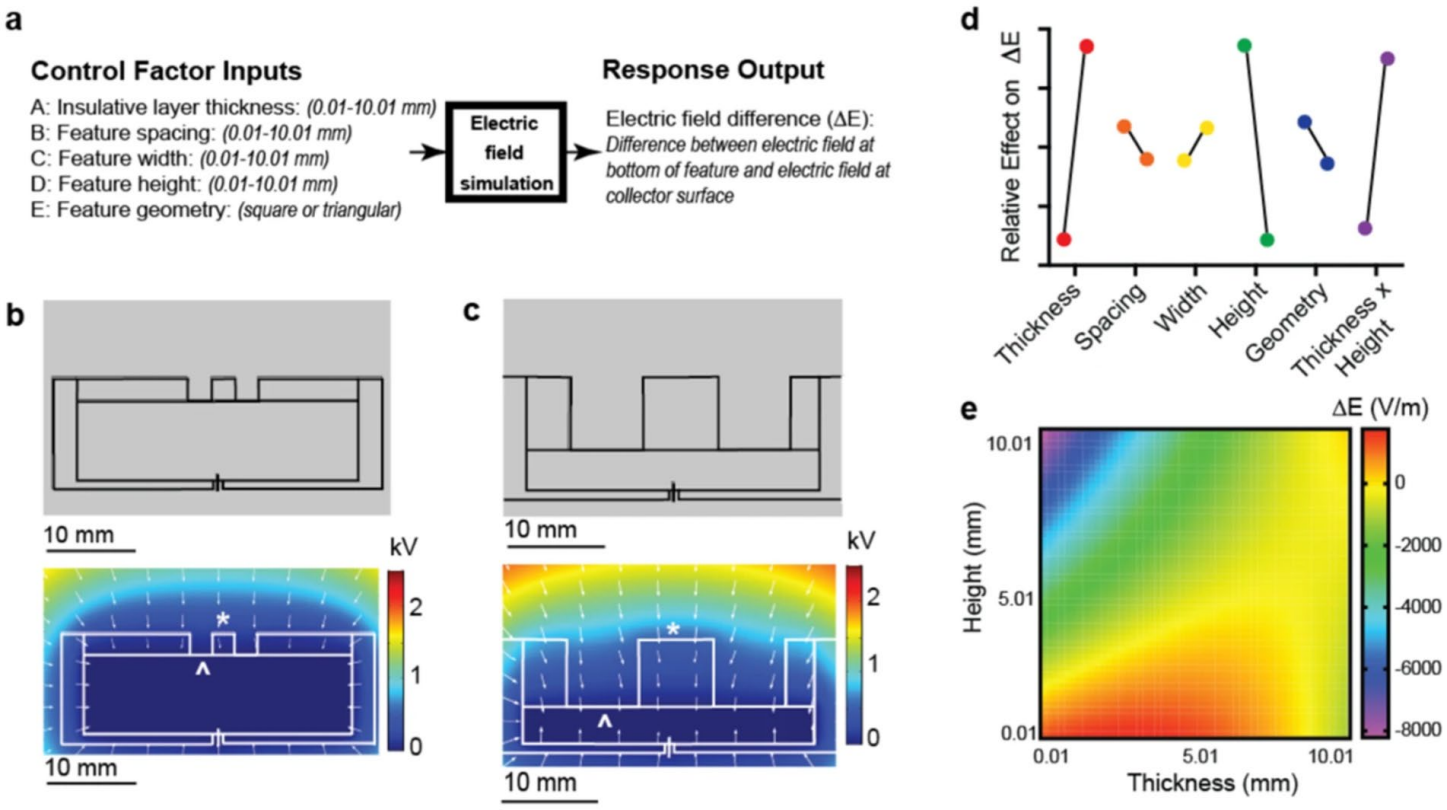
.

**Table 1:** Statistical analysis of finite element method simulations.

	Sum of squares	df	Mean square	F value	p-value, Prob > F
**Full factorial design**					
Model	6.05E7	6	1.00E7	228	1.37E-20
A-PDMS thickness	2.08E7	1	2.08E7	472	9.34E-18
B-Spacing	6.18E5	1	6.18E5	14.0	9.56E-04
C-Width	6.39E5	1	6.39E5	14.4	8.15E-04
D-Height	2.13E7	1	2.13E7	482	7.26E-18
E-Shape	8.59E5	1	8.59E5	19.4	1.70E-04
AD	1.62E7	1	1.62E7	367	1.81E-16
**Central composite design**					
Model	5.09E7	7	7.28E6	58.2	9.78E-13
A-PDMS thickness	1.32E7	1	1.32E7	105	7.18E-10
B-Spacing	1.91E5	1	1.91E5	1.53	0.229
C-Width	1.52E5	1	1.52E5	1.21	0.281
D-Height	2.70E7	1	2.70E7	216	7.16E-13
AD	7.13E6	1	7.13E6	57.0	1.51E-07
A^2^	3.12E6	1	3.12E6	24.9	5.29E-05
D^2^	162	1	162	0.00129	0.972

The correction of the error also necessitates textual changes, as below.

There was an error in the ‘Results and Discussion’ section, subsection ‘Electrospun fiber conformation to 3D collector pattern depends on insulative layer thickness and feature height’, where:

“PVA fibers deposited only on the surface of the fully conductive collector without the insulative layer, but fibers deposited densely in the patterns on the insulated two-layer collector (Fig. 4a,b). These results are in agreement with the simulations, which calculated a 365 V/m higher ΔE for the collector with the insulative PDMS layer.”

now reads:

“PVA fibers deposited only on the surface of the fully conductive collector without the insulative layer, but fibers deposited densely in the patterns on the insulated two-layer collector (Fig. 4a,b). These results are in agreement with the simulations, which calculated a 330 V/m higher ΔE for the collector with the insulative PDMS layer.”

There was another error in the same section and subsection where:

“The PDMS-based collectors were then scaled down by approximately 10-fold to verify that the patterning effect was valid at smaller length scales (conical pattern: 269 ± 5 μm diameter, 522 ± 6 μm height, 1400 μm spacing, 400 μm insulative PDMS layer thickness). Based on the simulations (ΔE = 882 V/m), we expected to observe fiber deposition in the patterns for this collector. We observed a similar patterning effect for this microscale collector, with fibers deposited densely within the patterns in 1–2 minutes and little to no deposition on the collector surface (Fig. 4c).”

now reads:

“The PDMS-based collectors were then scaled down by approximately 10-fold to verify that the patterning effect was valid at smaller length scales (conical pattern: 269 ± 5 μm diameter, 522 ± 6 μm height, 1400 μm spacing, 400 μm insulative PDMS layer thickness). Although the simulations calculated a ΔE value less than 0 V/m (− 119 V/m), we observed a similar patterning effect for this microscale collector, with fibers deposited densely within the patterns in 1–2 minutes and little to no deposition on the collector surface (Fig. 4c). These results suggest that experimental fiber patterning is possible at a wider range of ΔE values than those predicted by the simulations”.

Another error was in that subsection where:

“This collector contained conical patterns (364 ± 16 μm diameter, 777 ± 20 μm height, 1600 μm spacing), and an insulative PDMS layer that ranged from 400 to 580 μm. Our simulations calculated that the ΔE ranged from 647 V/m for the 400 μm PDMS thickness to 705 V/m for the 580 μm PDMS thickness.”

now reads:

“This collector contained conical patterns (364 ± 16 μm diameter, 777 ± 20 μm height, 1600 μm spacing), and an insulative PDMS layer that ranged from 400 to 580 μm. Our simulations calculated that the ΔE was 48 V/m higher for the 580 μm PDMS thickness compared to the 400 μm PDMS thickness.”

There were errors in the ‘Materials and Methods’ where:

“The results of the central composite design were used to construct a quadratic model (R^2^ = 0.9488) that can be used to predict ΔE for any given set of control factor inputs (Eq. 1).

$$ \Delta E = 1286 + \left( {293*thickness} \right) - \left( {35*spacing} \right) + \left( {31*width} \right) - \left( {963*height} \right) + \left( {106*thickness*height} \right) - \left( {53*thickness^{2} } \right) + \left( {0.382*height^{2} } \right)"$$

now read:

“The results of the central composite design were used to construct a quadratic model (R^2^ = 0.9958) that can be used to predict ΔE for any given set of control factor inputs (Eq. 1)”.

$$ \Delta E = 63 + \left( {231*thickness} \right) - \left( {35*spacing} \right) + \left( {31*width} \right) - \left( {455*height} \right) + \left( {106*thickness*height} \right) - \left( {46*thickness^{2} } \right) - \left( {42*height^{2} } \right)"$$

These errors have now been corrected in the PDF and HTML versions of the Article.

The overall conclusions of the Article are unaffected by these corrections.

