# Supplementary material for: In situ 3D-patterning of electrospun fibers using two-layer composite materials
Source: Sci Rep. 2020 May 14;10:7949. doi: 10.1038/s41598-020-64846-z (PMC7224382; doi:10.1038/s41598-020-64846-z)
Supplement: Supplementary file 1 — Supplementary Materials. [file 41598_2020_64846_MOESM1_ESM.docx]

**Title**

In situ 3D-patterning of electrospun fibers using two-layer composite materials

**Authors**

R. L. Creighton,^1^ J. Phan,^1^ K. A. Woodrow^1^*

**Affiliations**

^1^ Department of Bioengineering, University of Washington, Seattle, WA 98195 USA

*corresponding author

Email: woodrow@uw.edu

**Supplementary Methods**

*Preparation of drug loaded integrated fiber microneedles:* The electrospinning precursor solution was prepared by dissolving poly(L-lactide) (PLLA, Lactel Absorbable Polymers, ester terminated, inherent viscosity 0.9-1.2 dL/g) at 15% w/v in a 50:50 mixture of chloroform and hexafluoroisopropanol. Dapivirine (DPV, provided by the International Partnership for Microbicides) was then added to this solution at 30% (w/w) or 15% (w/w) relative to the mass of the polymer. The precursor solution was loaded in a glass syringe fitted with a 22G blunt tipped needle. The polymer solution was dispensed from the syringe using a syringe pump (New Era Pump Systems, Inc.) at a 1 μL/min flow rate. The microneedle mold collector (conical patterns with 300 μm diameter, 800 μm height, 1000 μm spacing, 500 μm insulative layer thickness) was fixed to a custom holder that inserted copper wires to the back of the collector and held the collector at the same height as the needle. The ground from the power source (Gamma High Voltage Research) was attached to the copper wires and the positive lead was attached to the base of the needle. A voltage of 17 kV was applied and the samples were electrospun for 30 seconds. After electrospinning, 100 μL of a 30% (w/v) solution of polyvinylpyrrolidone (PVP, average molecular weight 10 kDa, Sigma Aldrich) in water was added to the microneedle mold collector. The mold was then placed in a vacuum chamber for a total of 2 minutes. Then 100 μL of a 10% (w/v) solution of polyvinyl alcohol (product P1180, 85-89% hydrolyzed, Spectrum Chemical) in water was added, and the mold was placed in a vacuum chamber for one minute. After this vacuum step, an additional 100 μL of PVA solution was added to the mold. The microneedles were then dried at room temperature overnight.

*Preparation of drug loaded conventional matrix microneedles:* Drug loaded conventional matrix microneedles without fibers were prepared from a 15% (w/v) solution of PLLA in a 50:50 mixture of chloroform and HFIP without drug and with 15 wt% or 30 wt% dapivirine. 100 μL of this solution was applied to a microneedle mold (300 μm needle diameter, 800 μm needle height, 1000 μm needle spacing), then placed in a vacuum chamber for a total of 2 minutes. An additional 100 μL of the same polymer solution was added to the mold, followed by 1 minute of vacuum. Conventional matrix microneedles were dried at room temperature overnight, then under vacuum to remove residual solvent. Conventional matrix microneedles without drug were prepared by the same method.

*Dapivirine release:* Release studies were performed in phosphate buffered saline (pH 7.4) containing 1% cremophor. The volume of release media was adjusted to ensure sink conditions, defined as 10 times the solubility limit of dapivirine. Release was performed in a rotating shaker at 37ºC. At predetermined timepoints, 200 µL samples of release media were collected for HPLC analysis and replaced with an equal volume of fresh release media. Drug content was quantified using a Shimadzu Prominence UV-HPLC with a C18 column at 30ºC column temperature, 10 µL injection volume, 10 minute run time, and UV detection at 310 nm. A mixture of 65% acetonitrile and 35% 10 mM ammonium acetate in water was used as the mobile phase. A dapivirine standard curve was prepared in a 50:50 mixture of PBS and DMSO to enable quantification of drug in the release media. Percent release was calculated as the percent of the theoretically encapsulated drug.

**Supplementary Data**


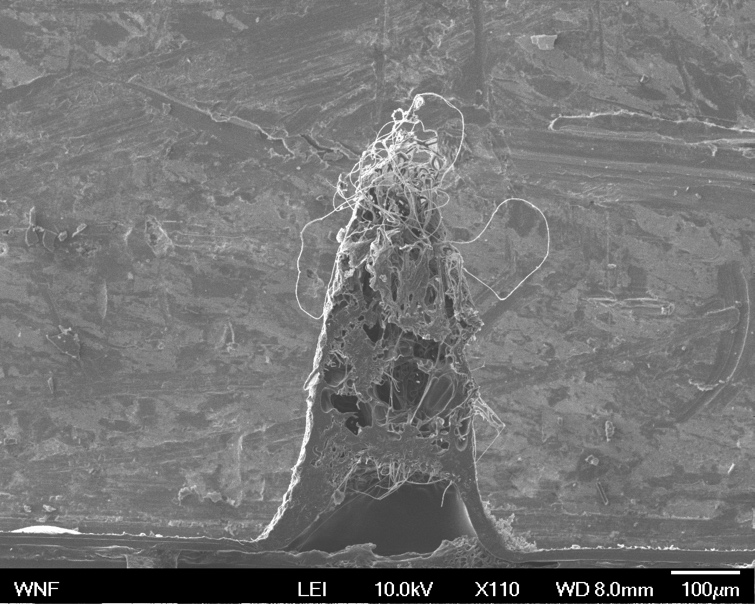


**Figure S1: Integrated fiber microneedles have a porous microarchitecture.** SEM imaging of an integrated fiber microneedle cross-section reveals an interconnected network of electrospun fibers (denoted by a black arrow) dispersed throughout the polymer matrix material (denoted by a white arrow).


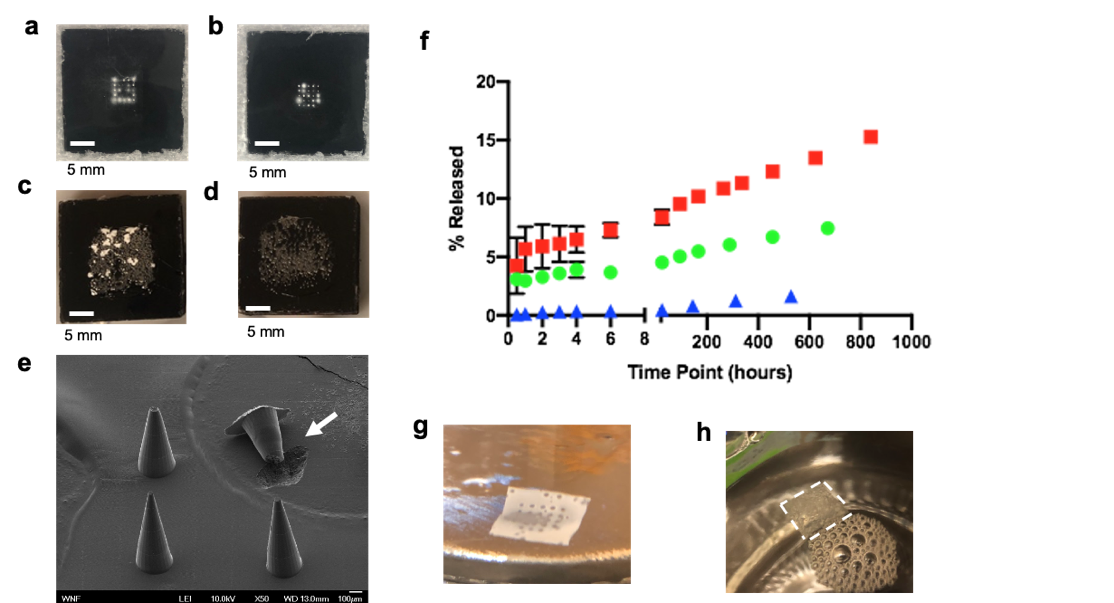


**Figure S2: Integrated fiber microneedles enable formulation and release of dapivirine.** PLLA fibers electrospun onto two-layer collectors (a) with and (b) without 30 wt% dapivirine. Preparation of PLLA microneedles (c) with 30 wt% dapivirine resulted in Ostwald ripening of the drug into large particles compared to (d) PLLA microneedles without drug. (e) SEM inspection of drug loaded PLLA microneedles revealed defects due to the particle formation (denoted by white arrow). (f) Percent dapivirine release over time was evaluated for integrated fiber microneedles at 15% (w/w) loading (green circles) and 30% (w/w) loading (red squares) and conventional matrix microneedles at 15% (w/w) loading (blue triangles). Data represents the mean±standard deviation of at least n=2 replicates. During the release experiment, (g) dapivirine loaded conventional matrix microneedles became opaque, while (h) conventional matrix microneedles without drug remained transparent. The boundary of the conventional matrix microneedles without drug is denoted with a white dashed line to improve visibility.

**Table S1: Electrospinning results for drug loaded and blank PLLA fibers**

| Dapivirine Loading (wt%)^a^ | Output (mg)^b^ | Yield (%)^b^ | S_E_ |
| --- | --- | --- | --- |
| 0 | 0.17 ^c^ | 96 | 4.88 |
| 15 | 0.15 ^d^ | 160 | 34.5 |
| 30 | 0.16 ^c^ | 71 | 1.35 |

^a^ All solutions were prepared at 15wt% PLLA, ^b^ Mean of at least n=2 electrospinning replicates, ^c^ Output is reported as total fiber mass on the collector after 1 minute or ^d^ 30 seconds of electrospinning
